# The *Ascosphaera apis* Infection (Chalkbrood Disease) Alters the Gut Bacteriome Composition of the Honeybee

**DOI:** 10.3390/pathogens12050734

**Published:** 2023-05-19

**Authors:** Dae Yoon Kim, Soohyun Maeng, Sung-Jin Cho, Hui Jin Park, Kyungsu Kim, Jae Kwon Lee, Sathiyaraj Srinivasan

**Affiliations:** 1 College of Pharmacy, Chungbuk National University, Chungbuk 28160, Republic of Korea; lnbsky@naver.com; 2 Department of Bio and Environmental Technology, College of Natural Science, Seoul Women’s University, Seoul 01797, Republic of Korea; 3 Department of Biological Sciences and Biotechnology, Chungbuk National University, Cheongju 28644, Republic of Korea; sjchobio@chungbuk.ac.kr (S.-J.C.);; 4 Department of Biology Education, College of Education, Chungbuk National University, Cheongju 28644, Republic of Korea

**Keywords:** honeybee, *Nosema ceranae*, *Apis mellifera*, *Nosemosis*, Chalkbrood, *Ascosphaera apis*

## Abstract

The declining honeybee populations are a significant risk to the productivity and security of agriculture worldwide. Although there are many causes of these declines, parasites are a significant one. Disease glitches in honeybees have been identified in recent years and increasing attention has been paid to addressing the issue. Between 30% and 40% of all managed honeybee colonies in the USA have perished annually over the past few years. American foulbrood (AFB) and European foulbrood (EFB) have been reported as bacterial diseases, Nosema as a protozoan disease, and Chalkbrood and Stonebrood as fungal diseases. The study aims to compare the bacterial community related to the *Nosema ceranae* and *Ascosphaera apis* infection on the gut of the honeybee and compare it with the weakly active honeybees. The Nosema-infected honeybees contain the phyla *Proteobacteria* as the significantly dominant bacterial phyla, similar to the weakly active honeybees. In contrast, the *Ascosphaera* (Chalkbrood) infected honeybee contains large amounts of *Firmicutes* rather than *Proteobacteria*.

## 1. Introduction

The majority of the 57 most essential crops for human consumption are produced by bee colonies, making them the most managed pollinators in the world [1,2,3]. The decline of the honeybee poses a serious threat to the productivity and stability of global agriculture. Parasites are indeed a significant one of the many causative factors of these declines. The honeybee gut bacteriome composition can vary, depending on various factors such as geography, diet, season, and management practices [4]. However, some common bacterial species found in the honeybee gut include *Lactobacillus*, *Bifidobacterium*, and *Gilliamella*. These bacteria play important roles in nutrient digestion and absorption, immune system regulation, and protection against pathogens [5]. Disease crises in honeybees have intensified in recent years and increasing attention is being paid to addressing the issue. Over the past several years, there have been annual losses of between 30% and 40% of all managed US honeybee colonies [6,7,8]. In addition to viruses, bacteria, microsporidia, and arthropods, a wide variety of parasites pose serious risks to honeybees [1,9,10].

The viral diseases that cause problems in bees include Kashmir bee virus, acute bee paralysis virus (ABPV), deformed wing virus (DWV), black queen cell virus (BQCV), acute bee paralysis virus (ABPV), and deformed wing virus (DWV) [11]. *Varroa destructor* is a parasitic mite that poses a significant threat to honeybee populations worldwide [12]. These mites feed on the hemolymph (the circulatory fluid) of both adult honeybees and developing broods, causing physical damage and weakening the bees [13]. The mite infestation not only leads to a decline in individual bee health, but can also facilitate the transmission of various honeybee viruses, exacerbating colony collapse [14]. American foulbrood (AFB) and European foulbrood (EFB) have been reported as bacterial diseases, *Nosema* as a protozoan disease, and Chalkbrood and Stonebrood as fungal diseases [15,16,17]. Among these diseases, *Nosemosis* and Chalkbrood disease are the most serious diseases in the Republic of Korea [18].

Honeybees rely on a diverse community of microbes to help with digestion, nutrition, and protection against pathogens. The developed intestine of a worker honeybee gut consists of four core bacterial symbionts that are mainly found in the hindgut: *Snodgrassella alvi*, *Gilliamella apicola*, *Lactobacillus*, and *Bifidobacterium asteroides* [5,19]. These constituents are acquired within the home colony and mainly established after 9 days of the bee emerging from the cell [20,21]. Other gut bacteria are frequent, but in relatively lower prevalence and with less consistency across bees and colonies. Many metabolic processes happen in the hindgut, especially in a symbiotic context. Of the core bacterial symbionts, *G. apicola*, *Bifidobacterium*, and *Lactobacillus* are carbohydrate fermenters whose metabolic products support *S. alvi* growth [22,23]. These symbiont-derived metabolites likely also support bee health via intestinal absorption. These findings were supported by microbial feeding studies focused on the physiological processes of bees, and metagenome annotations of the symbionts [4,24]. The midgut lies upstream of the hindgut. The honeybee midgut is the focal point of the digestion and absorption of nutrients. It possesses digestive enzymes from the cell lining, in addition to enzymes that are secreted and translocated from the hypopharyngeal glands [25,26]. The midgut is also lined with the peritrophic matrix, which is a protective barrier. Relative to the hindgut, the midgut is essentially void of bacteria, possibly due to the constant rearrangement of the peritrophic matrix [20]. The bee midgut is speculated to harbor yeasts that offer additional digestive roles, as is common in other insects [27]. This indicates that yeasts likely have an alternative method for the attachment/colonization in this environment that bacteria lack [22].

Since its discovery in 1909, *Nosemosis* has been viewed as a seasonal disease that has a negative impact on beekeeping profits. A microsporidian parasite called *Nosema ceranae* infects honeybees and can seriously harm honeybee colonies [28,29]. Research has shown that *Nosema ceranae* infection can alter the composition of the honeybee gut bacteriome, causing the abundance and the decline of beneficial bacteria such as *Lactobacillus* and an increase in potentially harmful bacteria such as *Enterococcus* [30,31]. In spring, many are exhausted and due to the effects of *Nosemosis*, dead bees are discovered all around the hives [32].

The most prevalent eukaryotic gut pathogen in honeybees is *Nosema ceranae*. Although infection is typically chronic, it can also be fatal. Recent research has linked the development of gut infectious diseases to the gut microbiota [21,33]. Interestingly, studies found positive, not negative, correlations between *Nosema ceranae* infection and the main bacteria in honeybee microbiota [34]. The gut microbiota of honeybees is basic and primarily made up of a few key bacterial species. There are not many gut bacteria in the midgut, possibly because of the peritrophic membrane’s ongoing regeneration [35,36,37].

A fungal pathogen *Ascosphaera apis* infects honeybee larvae, causing a disease known as Chalkbrood [38]. While research has shown that Chalkbrood can have significant impacts on a honeybee’s health, there is less information on the specific effects of *Ascosphaera apis* on the alteration of gut bacteriome composition in the honeybee [39,40]. Conversely, some studies have investigated the impact of Chalkbrood disease (caused by both *Ascosphaera apis* and *Ascosphaera larvis*) on the gut microbiome of honeybee larvae; for instance, Chalkbrood-infected larvae had reduced levels of certain beneficial bacterial species, such as *Lactobacillus* and *Bifidobacterium*, in their gut compared to healthy larvae [40,41,42]. The study also found an increase in the abundance of potentially harmful bacteria such as *Enterobacter* and *Pseudomonas* in Chalkbrood-infected larvae [43].

*Firmicutes* are gram-positive bacteria that include a diverse group of bacteria, some of which are commonly found in the honeybee gut [44]. They are known to play important roles in nutrient digestion, immune system regulation, and protection against pathogens. Some common families of *Firmicutes* found in the honeybee gut include *Lactobacillaceae*, *Bacillaceae*, and *Streptococcaceae* [44,45,46]. Studies have suggested that a higher relative abundance of *Firmicutes* is associated with better honeybee health and a greater resistance to diseases such as American foulbrood disease, while a higher relative abundance of *Proteobacteria* is associated with an increased susceptibility to certain diseases [5,47]. Several studies have suggested that *Lactobacillus* may play a protective role against *Nosema* and Chalkbrood infection in honeybees [48,49]. For example, a study by Rubanov et al. [34] suggested that honeybees with a greater relative abundance of *Lactobacillus* had a lower prevalence of Nosema infection. Similarly, [50] research work found that honeybees with a greater relative abundance of *Lactobacillus* had a lower prevalence of Chalkbrood infection.

Furthermore, *Nosema ceranae* infection has also been linked to changes in the gut microbiome diversity, which may have adverse effects on the health of honeybees generally and increase susceptibility to other illnesses. Understanding the interactions between *Nosema ceranae* infection and the honeybee gut bacteriome can help in developing effective strategies for the management and control of this parasite.

## 2. Materials and Methods

### 2.1. Experimental Design and Honeybee Collection

All the honeybees used in the experiment were purchased from the experiential colonies of an apiary located near Chungbuk Province, South Korea. Three different source colonies were used to collect the bees, which were then carefully transferred into mesh cages measuring 16.5 by 16.5 by 48 inches and kept at 24 ± 1 °C until transferred to the laboratory for dissection. The honeybees were maintained in artificial conditions for a period of up to 12 h prior to dissection. During this time, they were fed a diet of 50% sucrose solution, which was prepared using sterile distilled water, and provided with water ad libitum. The honeybee gut contents were collected from four different groups: control, *Nosema*-infected, Chalkbrood-infected, and weak bees. The bees were approximately 10 to 15 days old. Approximately 10 to 15 honeybees were selected randomly from each group and the gut contents were dissected and pooled for each sample. We conducted our analyses using five replicates per group, except for the Chalkbrood group, which contained four replicates. The weak group included bees collected from hives inhabited by flightless, floor-crawling bees. In general, a characteristic of beehives inhabited by weak bees is that there is little or no honey inside the hive.

The bees suffering from *Nosema* or Chalkbrood disease were confirmed through experimental methods and used as an experiment group for diseases. Nosema infections were diagnosed by a microscopic observation method of the Nosema spore. Nosema-infected bees contain spindled-shaped spores in the midguts. Bees with at least 1 × 10^6^ spores were classified as an experimental group infected with Nosema. Chalkbrood disease can be easily diagnosed using visual detection methods. Hives infected by Chalkbrood disease symptom appeared to have hard, shrunken chalk-like mummies in the brood and surrounding the entrance to the hive [38,51].

A molecular biology technique (Polymerase Chain Reaction, PCR) was used to differentiate the healthy condition of the honeybee [52,53]. *Nosemosis* was identified using previously described PCR methods with specific primers for *N. ceranae* (sense strand: 5′-CGG ATA AAA GAG TCC GTT ACC-3′, antisense strand: 5′-TGA GCA GGG TTC TAG GGAT-3′) and *N. apis* (sense strand: 5′ CCA TTG CCG GAT AAG AGA GT 3′, anti-sense strand: 5′ CAC GCA TTG CTG CAT CAT TGAC 3′) (Bioneer Co., Daejeon, Republic of Korea). Each PCR was preheated to 94 °C for 2 min, followed by 94 °C for 15 s, 60 °C for 30 s, and 72 °C for 45 s, with a final extension phase at 72 °C for 7 min. Chalkbrood disease (*Ascosphaera apis*) can also be easily diagnosed using the PCR method with specific primers [39,54,55] (sense strand: 5′-ACT CC CAC CCT TGT CTA CCT TA-3′, antisense strand: 5′-TCT TCG ACT GGA GTT CGT TTA TCT-3′) (Bioneer Co., Daejeon, Republic of Korea). Each PCR was preheated to 94 °C for 2 min, followed by 95 °C for 15 s, 60 °C for 30 s, and 72 °C for 45 s, with a final extension phase at 72 °C for 7 min. A variable number of cycles was used to ensure that the amplification occurred in the linear phase. The PCR products were separated on a 1.5% agarose gel and visualized by ethidium bromide staining and UV irradiation.

### 2.2. Chalkbrood (CB) Screening

The genomic DNA for CB screening was extracted from whole-bee homogenate aliquots using the DNeasy Plant Mini Kit (Qiagen, Hilden, Germany) according to the manufacturer’s protocol [27]. An assessment of the DNA yield and purity was performed using the NanoDrop 1000c spectrophotometer (Thermo Fisher Scientific, Waltham, MA, USA). The amplification of the internal transcribed spacer (ITS) region within the nuclear ribosomal repeat unit of the fungus *Ascosphaera apis* [56], and RpS5 gene of *A. mellifera* was completed using PCR. All PCR amplifications were performed using 2× Taq PCR MasterMix (abm, Richmond, BC, Canada), in 25 µL reactions, containing 400 nM of each primer, targeting either *A. apis* ITS or *A. mellifera* RpS5. The PCR conditions were as follows: 94 °C for 10 min; 30 cycles of 94 °C for 45 s, 62 °C for 45 s, and 72 °C for 1 min; and 72 °C for 5 min [25]. The PCR product evaluation was performed as above.

### 2.3. DNA Isolation and Sequencing

The metagenomic DNA was isolated from 10 g of homogenized gut content with the previously reported method [57,58,59]. The modifications were carried out by adding an enzymatic digestion (lysozyme and achromopeptidase) step before the SDS lysis and lowering the lysis temperature (55 °C instead of 65 °C). Subsequently, the DNA was purified in two agarose gel electrophoresis steps, first using 0.7%, and then in 1% agarose. The pure DNA was recovered from the gel with an agarose gel extraction kit (Roche). The quality of the preparations was assessed spectrophotometrically on NanoDrop ND-1000 (NanoDrop, Wilmington, DE, USA). Samples were preserved at −80 °C for future analyses.

### 2.4. 16S rRNA Amplification and Sequencing

The PCR amplification of bacterial 16S rRNA hypervariable region V3-V4 was carried out using primers 341F (CCT ACG GGN GGC WGC AG) and 805R (GAC TAC HVG GGT ATC TAA TCC). The V3-V4 region has been accepted as a low error-prone region for taxonomic assignment and community clustering [60,61]. The PCR was carried out by 30-s initial denaturation at 98 °C, 30 cycles of 10-s denaturation at 98 °C, 30-s annealing at 55 °C, 30-s elongation at 72 °C, and a 5 min final extension at 72 °C. The sequencing procedure was carried out using Illumina (Illumina, San Diego, CA, USA). The library was prepared by a standard library construction protocol (https://support.illumina.com/downloads/16s_metagenomic_sequencing_library_preparation.html (accessed on 12 December 2022)) by the Nextera XT kit (Illumina, San Diego, CA, USA), following the manufacturer’s instructions. The specific amplicons for the V3-V4 region were quantified in each reaction mixture and Illumina sequence adapter. The index primers (Nextera XT Index kit) were used in emulsion PCR to generate amplicon libraries, followed by a PCR clean up. The MiSeq libraries were quantified and then subjected to 300-nucleotide paired-end multiplex sequencing on an Illumina MiSeq sequencer.

### 2.5. Sequencing Data Analyses

The Illumina MiSeq sequencer produced demultiplexed (PE) raw reads, the quality of the reads was accessed by FastQC [62] and timed using Trimmomatic [63]. The filled demultiplexed reads were imported to the quantitative insights into Microbial Ecology 2 (QIIME2) for further analysis. The quality filtering, trimming, and denoising were performed using q2-dada2 [64]. The read dereplications, learning of the error rates, and the sample sequence variant inference with samples were performed using DADA2. The amplicon sequence variant (ASV) table and the removal of chimeras were performed using DADA2, followed by the taxonomy assignment and species assignment using the DADA2 and the SILVA v138.1 database ([65] accessed on 15 August 2022). The bacterial richness and diversity were analyzed using alpha and beta diversity matrices and indices such as Observed feature, Shannon index, Chao index, ACE index, Rarefaction curves, Weighted and Unweighted Unifrac distance matrices, and PcoA plots. GraphPad Prism 8 was used to perform a statistical analysis of the results obtained.

## 3. Results and Discussion

### 3.1. Microbial Symbiosis in the Honeybee Gut

**Figure 1:**
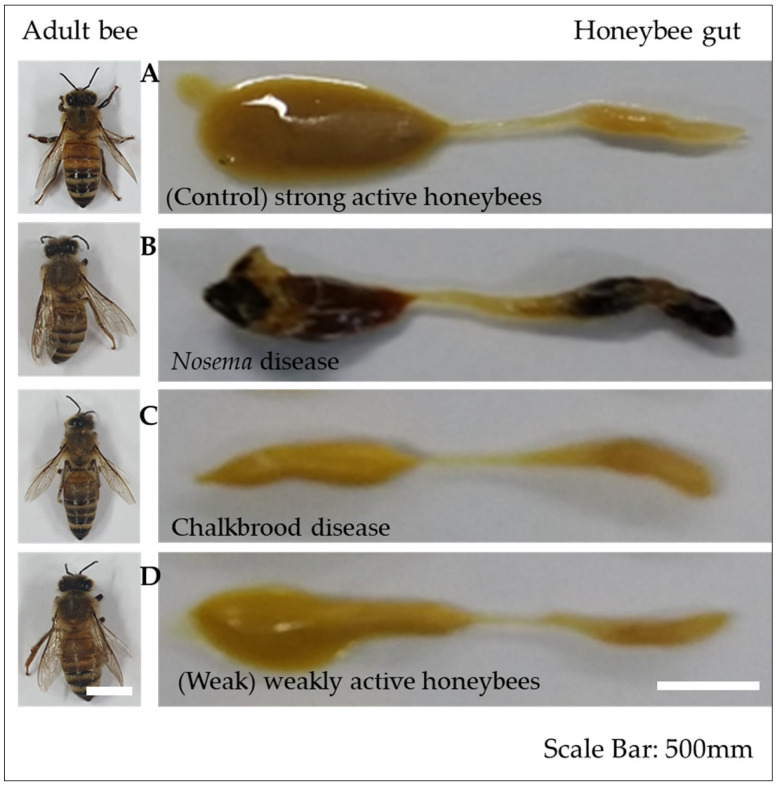
The dissection of the digestive tract of (**A**) control, (**B**) Nosema, (**C**) Chalkbrood, and (**D**) weak honeybees. The honeybees are dissected, and the gut contents were used for the analysis.

Microbial symbiosis plays a vital function in the gut of honeybees. The gut contents of the honeybee used in the experiment are shown in Figure 1. The results of the microbial symbiosis in the gut of honeybees revealed that the gut microbiota plays a crucial role in the digestion, immunity, and overall health at both the phylum and genus levels. The gut content of the control bees supports the beneficial bacterial growth, because of the mild acidity (pH 6.0–6.5) and the presence of enzymatic activities [4], which signify efficient nutrient breakdown and absorption [24]. The gut microbiota of honeybees consists of a core set of bacterial species, primarily belonging to the phyla *Proteobacteria* and *Firmicutes* [4,5]. This, in turn, supports overall bee health and colony well-being.

### 3.2. Taxonomic Analysis for Sequencing Data

Sequences generated for the polluted and control samples were analyzed using QIIME2 tools generating 1,353,431 total frequencies with an average of 42,294 OUT per sample. The taxonomic positions of sequenced reads were analyzed and studied using SILVA classifier, with classification based on 16S rRNA gene sequences. The analysis proposes that 98% of the reads belonged to the bacterial kingdom; other reads were omitted from further analysis. Since 16S rRNA is widely used for taxonomic and phylogenetic studies due to its highly conserved sequences, its hypervariable region can also be used for accurate taxonomic evaluation.

Honeybees infected with *Nosema ceranae* preferred sunflower honey over honeydew honey in dual-choice tests; sunflower honey had higher antimicrobial activity and decreased the amount of *N. ceranae* spores in the bee gut [56]. The gut of honeybees is home to bacteria that are antagonistic to parasites such as *Ascosphaera apis* [66,67]. Such antagonistic interactions might offer a means of treating diseases. For instance, the inoculation of bee colonies with the bacterium *Parasaccharibacter apium* resulted in the decreased levels of *Nosema ceranae* infection [68].

### 3.3. The Microbiome of Infected Honeybees

**Figure 2:**
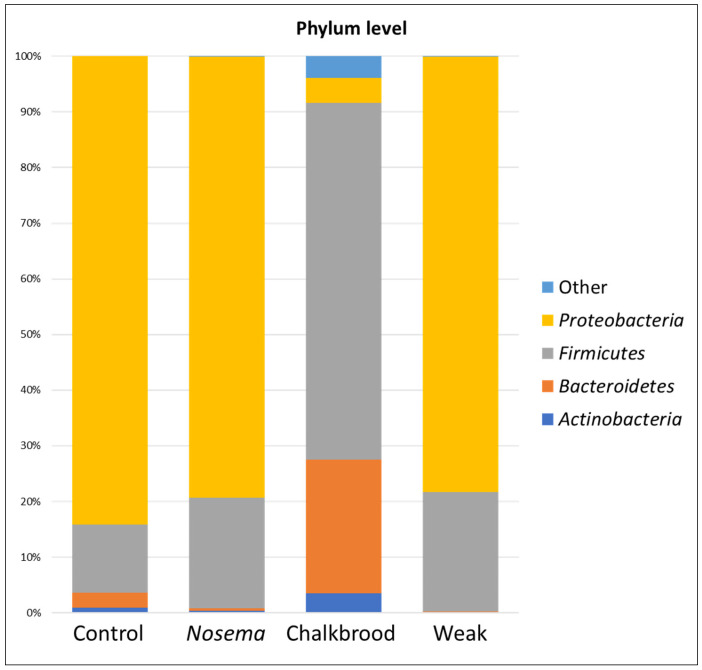
The phylum-level comparison between the *Nosema*, Chalkbrood, and weak honeybee gut bacterial communities.

**Figure 3:**
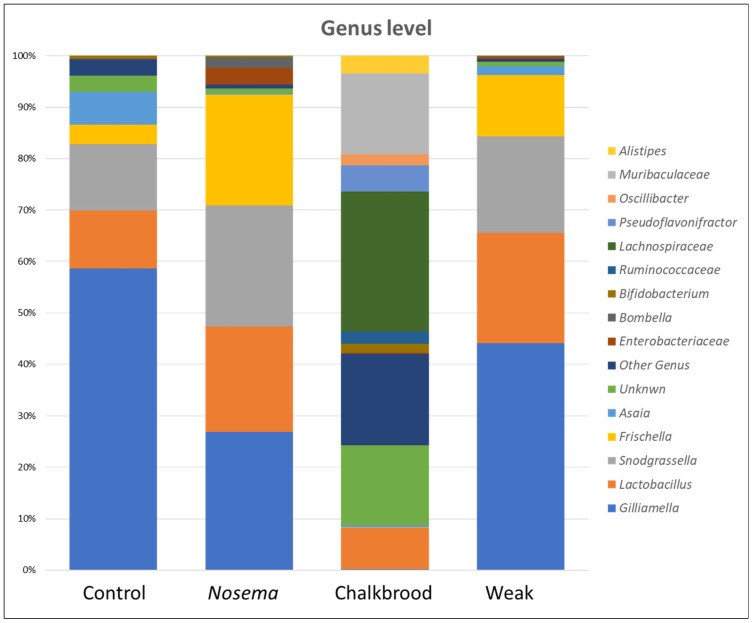
The genus level comparison between the *Nosema*, Chalkbrood, and weak honeybee gut bacterial communities.

The major bacterial community are from the phyla *Proteobacteria* and *Firmicutes* (Figure 2 and Supplementary Table S1), which are significantly different from the Chalkbrood honeybee’s guts. The bacterial species belonging to the genus *Gilliamella*, *Lactobacillus*, *Snodgrassella*, *Frischella*, and *Bombella* are predominantly present in the gut of *Nosema*-infected honeybees (Figure 3).

**Figure 4:**
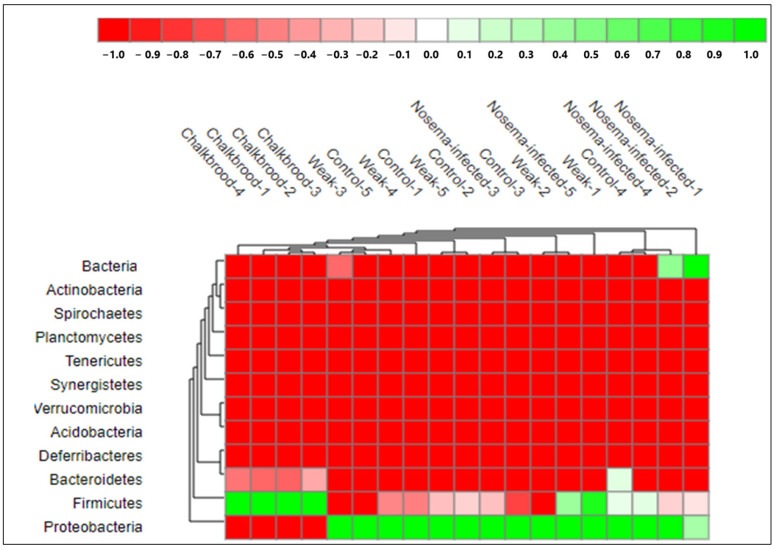
A heatmap of bacterial diversity associated with the *Nosema*, Chalkbrood, and weak honeybee intestine compared with the healthy control.

*Proteobacteria* and *Firmicutes* are two of the major phyla of bacteria found in the honeybee gut bacteriome. Together, they often make up the majority of bacterial species present in the honeybee gut. The heat-map of the response of bacterial community structure at the phylum level (Figure 4) shows the diversity of bacteria harbored in four different gut samples of the honeybee. *Proteobacteria* are gram-negative bacteria that include a diverse group of bacteria with various metabolic capabilities. They play important roles in nutrient digestion, immune system regulation, and protection against pathogens. Some common families of *Proteobacteria* found in the honeybee gut include *Acetobacteraceae*, *Enterobacteriaceae*, and *Pseudomonadaceae*.

**Figure 5:**
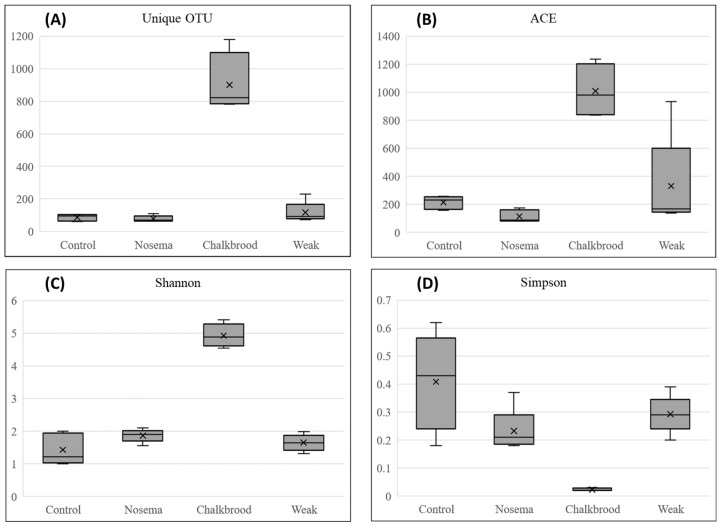
The comparison of alpha diversity indices (**A**) observed OTUs, (**B**) ACE index, (**C**) Shannon diversity index, and (**D**) Simpson index among four different groups. The Chalkbrood-infected honeybee gut shows significant differences among the groups.

Based on the alpha divert index (Figure 5 and Supplementary Table S3), the Chalkbrood-infected honeybee gut harbors significantly higher OTUs compared with the other three groups, and also shows high diversity of bacteria compared with other groups.

**Figure 6:**
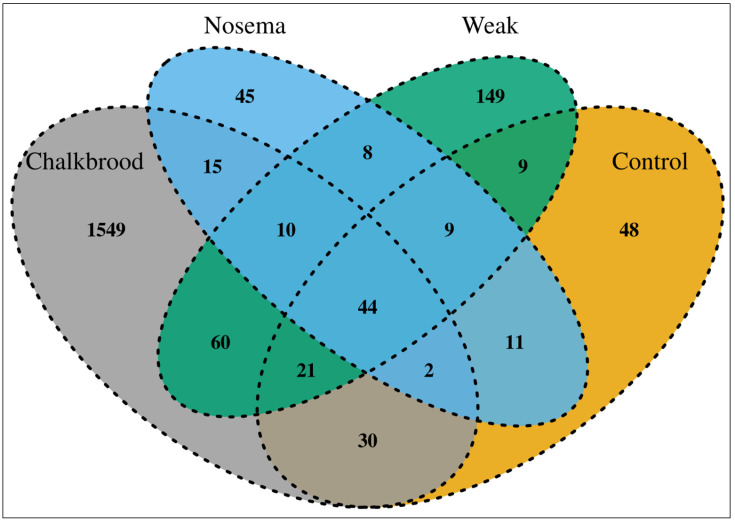
A four-way Venn diagram illustrating the number of shared unique OTUs at the 0.03 cut-off level between the honeybee gut samples. The Chalkbrood samples have the highest OTU diversity and the highest number of unique OTUs.

The bacterial diversity is significantly higher in the Chalkbrood-infected honeybee gut compared with other honeybees. The composition of a healthy Nosema and weak honeybee microbiome are significantly lower than the Chalkbrood-infected honeybee, which is also observed in the Shannon and Simpson diversity index. The Venn diagram shows the shared microbial community between the samples (Figure 6). The Nosema-infected honeybee harbors the lowest number of unique and total OTUs compared to other groups.

The Chalkbrood-infected honeybee displayed the most unique bacterial OTUs compared with the Nosema, weak, and control honeybee. The gut of the control honeybee shows the dominant presence of *Gilliamella*, *Lactobacillus*, *Frischella*, *Snodgrassella*, and *Asaia*, whereas the Nosema-infected honeybee showed the presence of *Gilliamella*, *Lactobacillus*, *Frischella*, *Snodgrassella*, *Commensalibacter*, *Franconibacter*, and members of the family *Enterobacteriaceae* (Figure 1 and Supplementary Table S2). The weak honeybee showed the presence of the genera *Gilliamella*, *Lactobacillus*, *Frischella*, *Snodgrassella*, *Pantoea*, and the members of the family *Enterobacteriaceae*; meanwhile, the Chalkbrood-infected honeybee shows the presence of *Lactobacillus*, *Pseudoflavonifractor*, *Alistipes*, *Oscillibacter*, *Paenibacillus*, and three unknown bacterial genera as the dominant genera.

## 4. Conclusions

The primary focus of the study was to analyze and contrast the bacterial communities present in the gut of honeybees infected with *Ascosphaera apis*, and to compare these findings with those from *Nosema ceranae*-infected and weakly active honeybees. In the case of *Nosema*-infected honeybees, the *Proteobacteria* phylum was found to be significantly dominant within the bacterial community, a characteristic that was also observed in weakly active honeybees. However, when examining the honeybees infected with *Ascosphaera* (Chalkbrood), the bacterial composition diverged considerably, as these bees were found to harbor substantial quantities of *Firmicutes*, rather than *Proteobacteria.*

The genus *Lactobacillus* is present predominantly in all four groups of honeybee guts, regardless of infection or weakness. The genera *Gilliamella*, *Frischella*, and *Snodgrassella* are present in Nosema, weak, and control honeybee guts, but not significantly present in the Chalkbrood-infected honeybees. This shows that these three genera are significantly affected by the *Ascosphaera apis* infection in honeybees. Studies have shown that honeybees infected with *Nosema ceranae* have a reduced gut bacteriome diversity and altered microbial community structure. Specifically, the prevalence of specific bacterial taxa, such as *Lactobacillus* and *Bifidobacterium*, has been found to decrease in infected bees. These bacteria are important for maintaining a healthy gut environment and aiding in digestion. The decrease in gut bacteriome diversity and altered microbial community structure may have negative consequences for the health and survival of honeybees. For example, a disrupted gut bacteriome can make honeybees more susceptible to other pathogens and environmental stressors.

The genus *Lactobacillus* is consistently found in the honeybee gut across all four groups, regardless of infection status or weakness [49]. Moreover, the genera *Gilliamella*, *Frischella*, and *Snodgrassella* are observed in the guts of *Nosema*-infected, weak, and control honeybees, but they are not significantly present in Chalkbrood-infected honeybees. This finding suggests that these three genera are considerably impacted by *Ascosphaera apis* infections in honeybees [49]. The research has demonstrated that honeybees infected with *Nosema ceranae* exhibit a reduced gut bacteriome diversity and altered microbial community structure [2]. Specifically, the prevalence of certain bacterial taxa, such as *Lactobacillus* and *Bifidobacterium*, decreases in infected bees. These bacteria are vital for maintaining a healthy gut environment and supporting digestion. The decline in gut bacteriome diversity and the altered microbial community structure may negatively affect the health and survival of honeybees. For instance, a disrupted gut bacteriome can increase honeybees’ susceptibility to other pathogens and environmental stressors.

More research is required to understand the specific roles of these bacteria in the gut. The *Ascosphaera apis* infection in the honeybee satirically alters the bacterial community structure in the gut, which can also be confirmed by the beta-diversity analysis (Supplementary Figures S1 and S2) with a UPGMA phylogenic tree and PCoA analysis. Overall, the impact of *Nosema ceranae* and *Ascosphaera apis* infection on the gut bacteriome of honeybees highlights the complex and interconnected nature of microbial communities in honeybee health and emphasizes the importance of studying these interactions to better understand and protect honeybee populations.

## Data Availability

The amplicon sequencing raw data for this study is available in the NCBI’s Sequence Read Archive (SRA), under BioProject accession number PRJNA972619.

## Supplementary Materials

The following supporting information can be downloaded at: https://www.mdpi.com/article/10.3390/pathogens12050734/s1. Supplementary Table S1: The bacterial community composition at the phylum level. Percent relative abundances of bacterial taxa at the phylum level are shown for samples. Relative abundances < 1% across all samples are shown as “Others”; Supplementary Table S2: The bacterial community composition at the genus level. Percent relative abundances of bacterial taxa at the genus level are shown for samples. Relative abundances < 1% across all samples are shown as “Others”; Supplementary Table S3: Alpha-diversity indices; Supplementary Figure S1: UPGMA phylogenetic tree based on weighted unifrac distance across the samples. Sequences were evenly pooled across individual samples (35,000 sequences per sample) prior to analysis; Supplementary Figure S2: The PCoA plot of beta diversity of the gut bacteriome based on weighted Unifrac distances of the honeybees. Each data point represents an individual sample. Sequences were evenly pooled across individual samples (35,000 sequences per sample) prior to analysis.
