# Supplementary material for: The Ascosphaera apis Infection (Chalkbrood Disease) Alters the Gut Bacteriome Composition of the Honeybee
Source: Pathogens. 2023 May 19;12(5):734. doi: 10.3390/pathogens12050734 (PMC10224485; doi:10.3390/pathogens12050734)
Supplement: Supplementary file 1 [file pathogens-12-00734-s001.zip › Supplementary File.pdf]

**Supplementary File**

*Article*

# The *Ascosphaera apis* Infection (Chalkbrood Disease) Alters the Gut Bacteriome Composition of the Honeybee

**Dae Yoon Kim [1,†], Soohyun Maeng [2,†], Sung-Jin Cho [3,†], Hui Jin Park [4], Kyungsu Kim [3], Jae Kwon Lee [4,*] and Sathiyaraj Srinivasan [2,*]**

1 College of Pharmacy, Chungbuk National University, Chungbuk 28160, Republic of Korea; lnbsky@naver.com
2 Department of Bio and Environmental Technology, College of Natural Science, Seoul Women's University, Seoul 01797, Republic of Korea; sarahmaeng@gmail.com
3 Department of Biological Sciences and Biotechnology, Chungbuk National University, Cheongju 28644, Republic of Korea; sjchobio@chungbuk.ac.kr (S.-J.C.)
4 Department of Biology Education, College of Education, Chungbuk National University, Cheongju 28644, Republic of Korea
* Correspondence: chemokine@chungbuk.ac.kr (J.K.L.); drsrini@swu.ac.kr (S.S.)
† These authors contributed equally to this work.

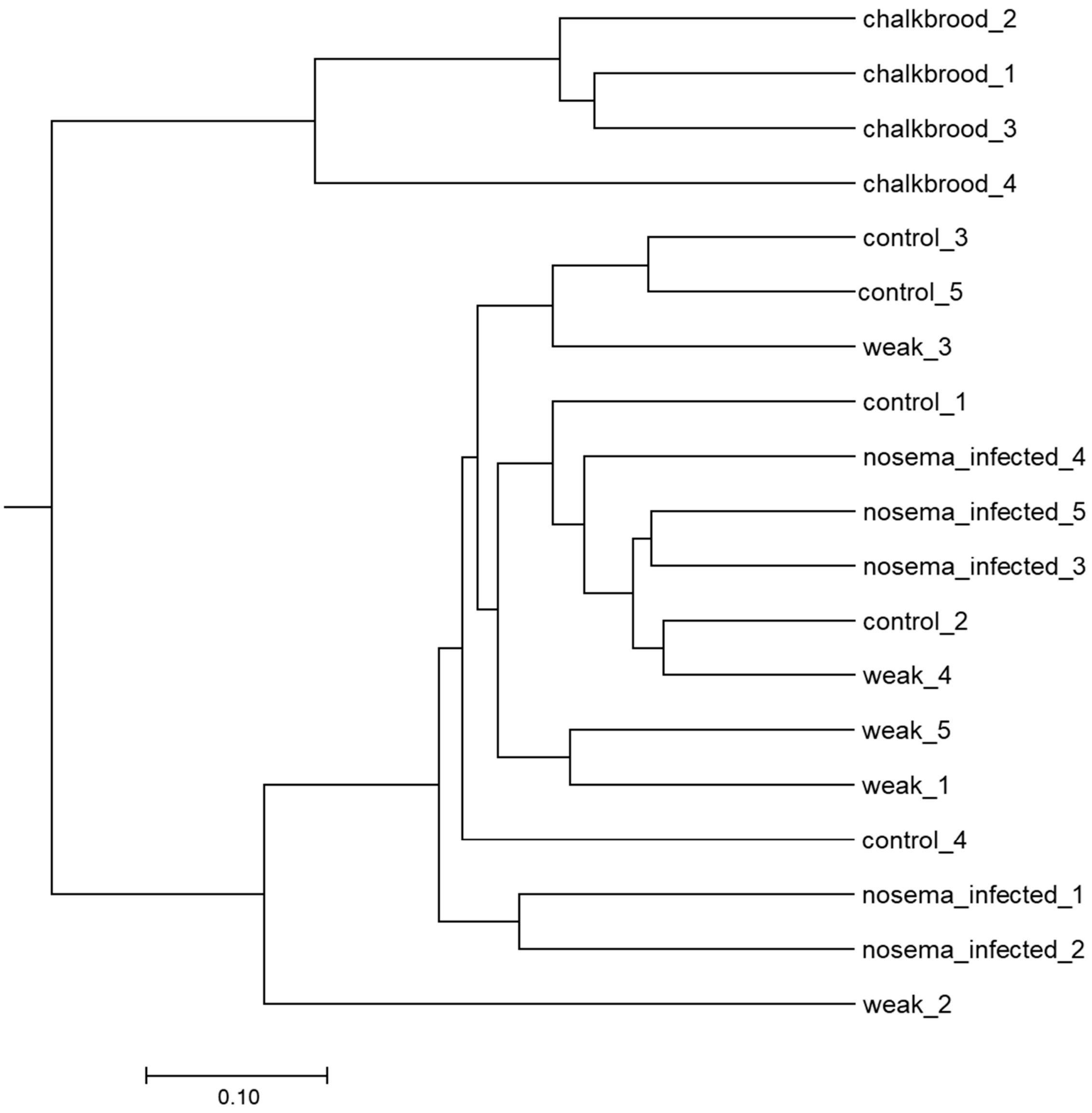


**Supplementary Figure S1**. UPGMA phylogenetic tree based on weighted unifrac distance across the samples. Sequences were evenly pooled across individual samples (35,000 sequences per sample) prior to analysis.

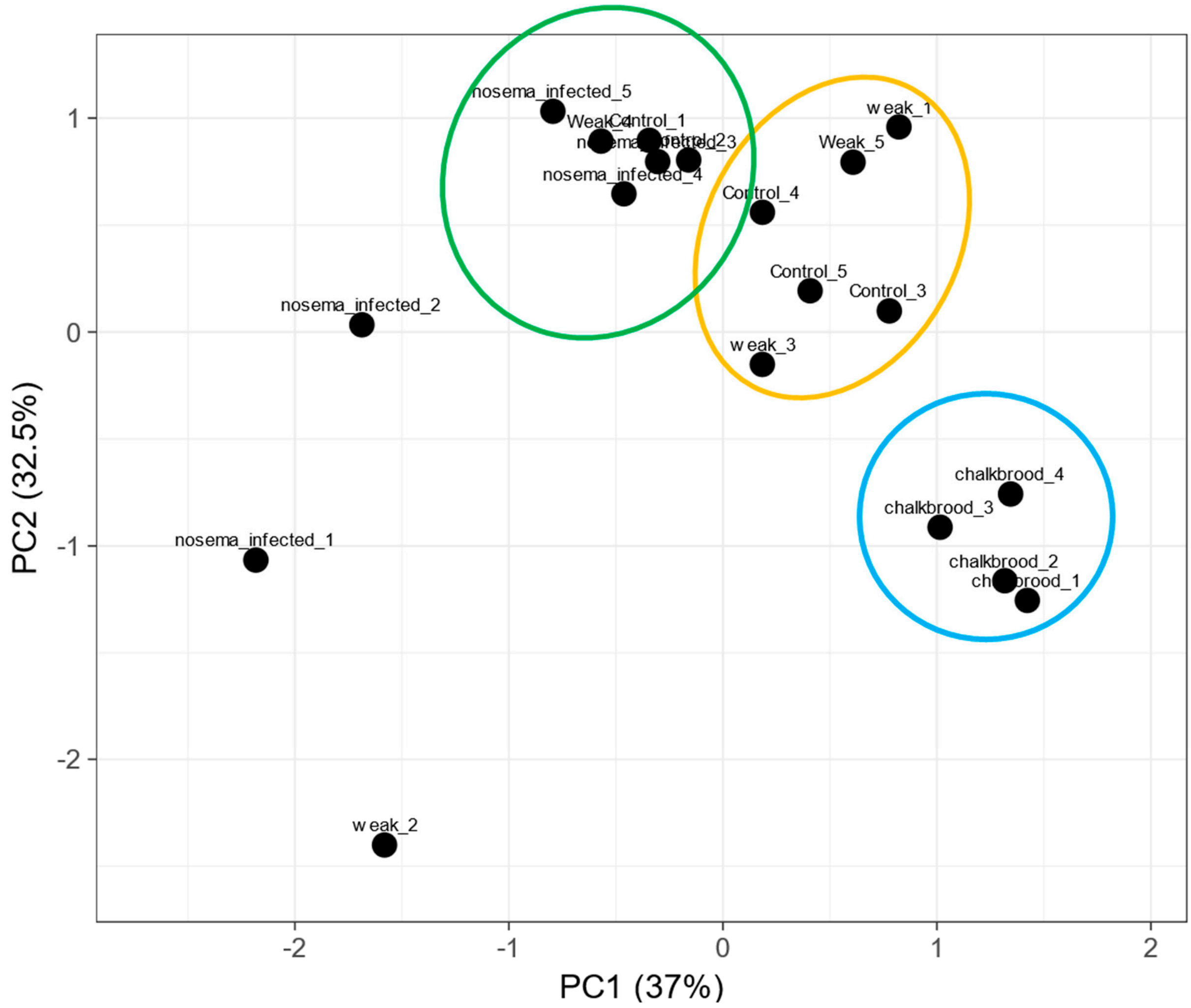


**Supplementary Figure S2**. The PCoA plot of beta diversity of the gut bacteriome based on weighted Unifrac distances of the honeybees. Each data point represents an individual sample. Sequences were evenly pooled across individual samples (35,000 sequences per sample) prior to analysis.
